# Three-dimensional geometric morphometric shape analysis of chest wall kinematics in different breathing conditions

**DOI:** 10.1098/rsos.240548

**Published:** 2024-07-17

**Authors:** Marta Gómez-Recio, Markus Bastir, Antonella LoMauro, Andrea Aliverti, Benoit Beyer

**Affiliations:** ^1^ Departamento de Paleobiología, Museo Nacional de Ciencias Naturales, CSIC, Madrid, Spain; ^2^ Dipartimento di Elettronica, Informazione e Bioingegneria (DEIB), Politecnico di Milano, Milano, Italy; ^3^ Laboratory for Functional Anatomy, Faculty of Motor Sciences, Université libre de Bruxelles, Bruxelles, Belgium

**Keywords:** breathing kinematics, optoelectronic plethysmography, geometric morphometrics, posture, exercise, chest wall

## Abstract

Breathing motion is based on the differential activity of the thoracic, diaphragmatic and abdominal muscles. Muscle contributions differ between rest and exercise conditions and depend on posture and other factors. Traditionally, these changes are investigated on volumetric data using optoelectronic plethysmography (OEP). OEP offers insight into size variations of different chest wall (CW) compartments but does not provide three-dimensional visualization methods of CW breathing kinematics. Here we explore the use of three-dimensional geometric morphometrics to analyse size and shape changes caused by spontaneous breathing motion during quiet (QB), and recovery breathing (REC, immediately after heavy exercise) in two different postures (SIT, sitting on cycle ergometer; STA, standing position). Our findings show that size and shape differ significantly between inspiration and expiration and that differences are greater in REC than in QB. However, this is achieved by stronger expiration in SIT but by greater expiratory and inspiratory movements in STA. Shape analysis suggests that these differences may be attributed to constrained mobility of the shoulder girdle and a minor thoracic spine extension during inspiration owing to position on the ergometer. Breathing motion in STA seems biomechanically less constrained. Geometric morphometrics analyses can provide additional insights into data obtained by OEP.

## 1. Introduction

The chest wall (CW) is the external cover of the thorax and the abdomen. It plays an important role in breathing. It protects the lungs and expands by the same amount of lung volume variation. The skeletal support of the CW provides insertion sites for the respiratory muscles (namely, the rib cage muscles, the diaphragm and the anterior abdominal muscles) that directly act on it [1,2]. CW motion therefore provides information on respiratory muscles actions. At rest in an upright position, quiet breathing is usually achieved by more thoracic than abdominal expansion, related to a higher action of the inspiratory ribcage muscle compared with the diaphragm. During exercise, increasing energetic demands of the body require the recruitment of thoracic, diaphragmatic and abdominal breathing muscle contributions to breathing motion, which allow for greater tidal volumes. At rest, expiration is usually passive and driven by the elastic recoil. During exercise, expiration is active. Aliverti and collaborators observed that the expiratory reserve volume is entirely located in the abdomen as a consequence of the contraction of the abdominal muscles, while the inspiratory reserve volume is located in the thoracic compartments because of a significantly increased action of inspiratory ribcage muscles (and not of the diaphragm) [3]. Hence, CW motion shows greater overall morphological changes that occur both in its upper and lower regions.

Multiple studies addressed these questions in health and clinical backgrounds using optoelectronic plethysmography (OEP) [3–5]. OEP is a non-invasive motion capture method that provides the three-dimensional coordinates of passive reflecting markers put on the CW according to anatomical points. The combination of dedicated geometric models and coordinates provides the volume enclosed by the mesh of markers. This analysis of different volumetric contributions of the CW compartments to breathing kinematics can be performed in different modes of activity and different postures. The effect of prone, supine or various sitting positions are reported [4]; however, the literature concerning the difference between standing and sitting posture is scarce. No differences in thoracoabdominal motions have been found between standing and sitting, and the effect of exercise in these postures is unknown [6].

OEP provides precise volumetric measurements (size) of breathing kinematics but does not inform about three-dimensional shape kinematics of the CW. Nevertheless, a simultaneous quantification and (commensurable) visualization of changes in CW shape can contribute to improve anatomical interpretations of associated breathing biomechanics. Literature addressing the topic of trunk shape (sensu stricto (*s.s*.) [7]) analysis is scarce. Usually, studies rely on anthropometric measurements that can be characterized from a three-dimensional digital environment [8,9]. These studies analyse specific parameters of trunk shape, specific to their objectives of studies. However, only studies based on geometric morphometrics quantify overall trunk shape (*s.s*.) and apply the statistical analysis of shape [10–12].

Three-dimensional geometric morphometrics (3DGM) [7,13] provides a set of tools for analysing three-dimensional size and shape variation both quantitatively and visually. These methods involve the statistical analysis of specifically processed Cartesian three-dimensional coordinates of landmarks that are placed on biologically comparable anatomical locations of subjects in a given sample. Owing to the powerful, landmark-driven visualizations that are possible when used in a virtual environment [14], 3DGM offers new opportunities to identify and localize variations in size (volume) and shape that occur owing to breathing motion changes. This holds the potential for improving our understanding of respiratory motion and underlying muscle recruitment at specific landmark locations.

### 1.1. Aim of the study

Our aim was to apply the innovative toolkit of 3DGM to the OEP three-dimensional markers coordinates for separate analyses of CW size and shape related to different breathing kinematics, namely quiet (QB) and recovery breathing (REC) after submaximal exercise, and to body posture, namely seated and standing. We hypothesized that different breathing modes (QB versus REC) and postures (seated versus standing) are associated with different patterns of CW motion during breathing.

## 2. Material and methods

### 2.1. Sample

A total of 27 asymptomatic young male adults, between the ages of 18 and 34 were included in the groups of study (table 1) and grouped according to posture. The first group included 14 healthy volunteers who were acquired while sitting on the cycle ergometer (SIT). The second group included 13 subjects acquired in standing position (STA).

**Table 1.** Descriptive statistics of the groups.

sample/descriptive		age (yr)	body height (m)	body weight (kg)	BMI (kg m^−2^)	WR peak (W)
SIT	*N*	14	14	14	14	14
	min	20	175	60	19.6	200
	max	31	191	100	29.5	352
	mean	26.4	180	76.9	23.8	285.6
	s.d.	5.2	5	10.8	2.8	52.8
STA	*N*	13	13	13	13	13
	min	21	172	63	20.34	300
	max	30	188	115	33.13	420
	mean	25.93	179.43	83.34	25.89	357.86
	s.d.	2.87	6.2	15.13	4.5	43.18

Participants were excluded if they had a history of cardiovascular or respiratory disease or any abnormalities on the surface of the torso. The detailed descriptive statistics of both groups are shown in table 1.

The protocol and the procedures of the investigation were approved by the Comité d’Ethique Erasme-ULB and by the National Committee for Medical Ethics at the Ministry of Health of the Republic of Slovenia (NCT02293772), and they were performed in agreement with the recommendations set forth in the Helsinki Declaration.

### 2.2. Motion capture systems and data collection

**Figure 1.**
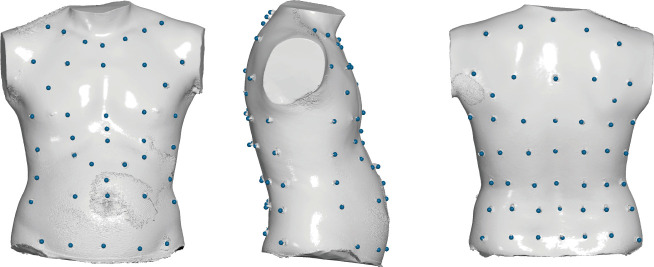
Template with the standard 89 markers in OEP analyses in anterior, right lateral and posterior views on a three-dimensional mesh obtained by a surface scan of the chest wall of a given volunteer.

The detailed protocol for the placement and recording of landmarks described by Cala *et al.* [15] was followed. It consists of placing 89 reflective landmarks on standardized specific anatomically homologous points on the CW surface (figure 1). All the trajectories of the 89 individual markers (defined by Cartesian coordinates) were recorded with a three-dimensional motion-capture stereophotogrammetry system (VICON 612, 14 cameras, data collection frequency: 100 Hz for STA; BTS OEP System, six cameras, data collection frequency: 60 Hz for SIT). System calibration was similar to the one used for standard gait analysis (volume dimension: 3 × 2 × 2 m). The high-resolution cameras were positioned at three different heights around the subject and tracked the movement of the reflective markers attached to the CW.

### 2.3. Experimental protocol

Before the experiment was conducted, the subjects were instructed to perform a steep ramp test. This is a short maximal exercise test that does not require respiratory gas analysis measurements. It was performed on a cycle ergometer during an incremental activity until exhaustion and its main outcome is the achieved peak work rate (WR_peak_). To achieve the WR_peak_, verbal encouragement was given by the technical team until the participant terminated the steep ramp test, either because of voluntary exhaustion or when their pedalling frequency fell below 60 r.p.m. [16].

To reduce the potential influence of conscious breathing, the volunteers were not informed about the aim of the study, which was to analyse breathing motion. The spontaneous quiet breathing was recorded for 10 min in a standing position for the STA group and seated on the bike for the SIT group. Afterwards, the subjects performed a constant effort between 50% and 60% of the maximum intensity (WR_peak_) while pedalling at a cadence between 60 and 70 r.p.m., which they maintained via visual and verbal feedback, for 5–6 min. Then, the subjects were asked to stop pedalling and to recover, the SIT group remained on the bike, while the STA group stood up beside the bike, with the hands placed on the hips (this helps the capture of the markers by the infrared cameras). In order to evaluate the effect of exercise on torso size and shape change, the breathing motion of the CW was then recorded for another 10 min.

**Figure 2.**
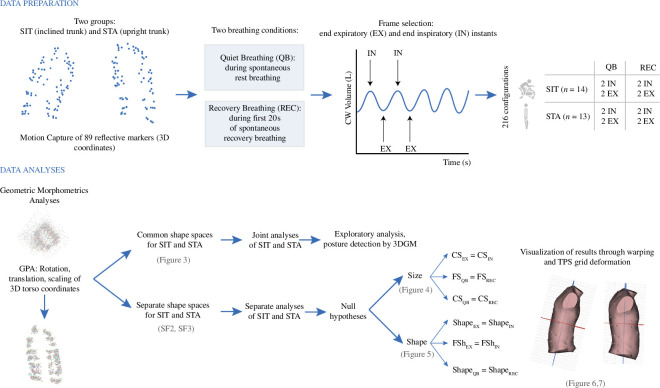
Complete workflow of data processing, null hypotheses and 3DGM analyses. The volumes of the chest wall were used to determine the endpoints (IN, EX) of respiratory cycles during QB and REC, and the 89 coordinates of the markers were extracted at these instants for morphometric analyses of respiratory shape kinematics. Analyses consisted of the application of generalized Procrustes analysis (GPA) algorithm to the variance–covariance matrix of the raw coordinates, first one GPA for SIT and STA together (exploratory analysis) and then one GPA for SIT and another for STA for the separate treatment of SIT and STA groups in the evaluation of hypotheses. QB: quiet breathing; REC: recovery breathing; CS: centroid size; FS: functional size; FSh: functional shape.

The volumes enclosed by the three-dimensional landmark configurations were computed and these signals were processed through the MATLAB environment [17]. To determine the end-inspiratory (IN) and end-expiratory (EX) instants of the breathing cycles, we selected two local peaks of maximum volume and two local peaks of minimum volume for each breathing condition in each of the 27 subjects (figure 2). This allowed the extraction of a total of eight CW three-dimensional-marker configurations per subject (SD1) through the Mokka software v. 0.6.1 [18].

### 2.4. Geometric morphometric procedures and variables

This work presents the first approach to respiratory motion analysis using the 3DGM toolkit. To do so, we converted the three-dimensional coordinates of the infrared markers’ position into three-dimensional Cartesian landmarks coordinates characterizing the configuration of the CW. The variations of the landmarks are related to the CW surface morphology of the subjects and the changes in CW shape during breathing motion and positions. Specifically, for this study, we considered only the CW landmark configurations that correspond to the instants of end-expiratory status (EX; detected by minimum CW volume) and end-inspiratory status (IN; detected by maximum CW volume).

These landmarks were then analysed by geometric morphometrics [7,13], which is a mathematical approach to measuring biological shape through statistical analysis of homologous points (landmarks) characterized by Cartesian coordinates (here, the three-dimensional coordinates of the infrared markers). 3DGM breaks down the variation of biological forms into the size and shape for separate analyses. The size is defined as centroid size and is the square root of the sum of squared distances between each landmark and the grand mean of all landmarks (= centroid). The shape is the residual information on the landmark coordinates after a generalized Procrustes analysis (GPA) [19] that standardizes by registration of the landmark data using translation, rotation and scaling. GPA first registers all landmark configurations to the first configuration and then, iteratively, on the means of these until the distance between corresponding landmark configurations approaches a minimum. Translation, rotation and scaling are considered invariant with respect to shape following Kendall’s definition of shape [20] and the GPA-registered coordinates represent the variation of pure shape. 3DGM not only allows for the statistical quantification of shape variation but also provides a detailed description of differences in shape in an intuitive graphic method based on the consistency and repeatability of the mathematical analysis of geometric variation (electronic supplementary material, figureS1). Isomorphy of the statistical and visualization spaces guarantees that the interpretation of the shapes corresponds totally to the statistical results [21].

Before the size and shape analyses were carried out the coordinates of some missing markers had to be estimated. This is a common problem in motion capture experiments that originates when the tracking system fails to detect the location of specific reflective markers over a period of time. Therefore, we estimated missing markers through the thin-plate spline method (TPS) [22] using *estimate.missing* function of geomorph R-package [14,23]. GPA was performed over a total of *n* = 216 landmark configurations that consisted of the EX and IN shape configurations of the 27 subjects in QB and REC breathing conditions in STA and SIT positions (SD1).

### 2.5. Statistical analyses and hypothesis framework

The first approach to the statistical treatment of the group was through a principal component analysis (PCA) over the variance–covariance matrix of the Procrustes coordinates (obtained after GPA of the whole sample). The purpose of this analysis was to reflect the shape variation of SIT and STA groups, independently of size. The PCA was performed in Kendall’s shape space to describe the postural, morphological and motion components that define the sample within a common shape space. This allowed a first description of the morphological variation and respiratory motion differences between postures. Owing to postural differences between groups leading to slightly different experimental designs between STA and SIT, two different PCAs were made to explore each group’s variability. For each PCA, mean respiratory vectors and their three-dimensional visualizations were computed. The following statistical analyses were carried out separately for both SIT and STA to test the following hypotheses. All hypotheses were tested through paired Wilcoxon signed-rank tests (non-parametric treatment of the data).

#### 2.5.1. Size hypotheses

Our first null hypothesis for size (H1_size_) predicts no differences between the medians of centroid size (CS) at EX and IN kinematic stages, neither during QB nor during REC. We expect to reject this null model by finding greater sizes at IN than EX stages owing to the inspired (tidal) air volume.

Our second null hypothesis for size (H2_size_) predicts no differences in size changes between QB and REC. We expect to reject this null model owing to greater tidal volumes necessary during exercise (REC) than during QB. To address H2_size_, that is, to test whether CS differences were greater in REC than in QB, we calculated ‘functional size, FS’, i.e. the difference in CS between IN and EX [24] in each subject and breathing condition. FS is a variable of net size change in a mean breathing cycle.

Our third null hypothesis for size (H3_size_) predicts no size differences between EX during QB and EX during REC as well as IN during QB and IN during REC. We tested this hypothesis in order to understand if differences in net size are caused by differences in the inspiration or the expiration. We expect to falsify this null model because the increase of tidal volume is achieved by greater expiration during exercise [1,3].

#### 2.5.2. Shape hypotheses

Our first null hypothesis for shape (H1_shape_) predicts no differences between CW shape at EX and IN kinematic stages, neither during QB nor during REC. We expect to reject this null model by finding a different shape at IN than EX stages owing to the inspired (tidal) air volume. This shape analysis compared the median scores of PC1 and PC2 of two different PCAs based on two different GPAs (one for the STA, PCA_STA_, and one for the SIT, PCA_SIT_, groups), to avoid potential effects of posture.

Our second null hypothesis for shape (H2_shape_) predicts no differences in shape changes between QB and REC. We expect to reject this null model owing to greater tidal volumes necessary during exercise (REC) than during QB. These should require greater shape changes owing to thoracic, diaphragmatic and abdominal respiratory muscle activity. To test H2_shape_, we extracted a Procrustes distance matrix from each GPA and computed the Procrustes distance between IN and EX for each subject and breathing condition, creating a new variable, functional shape (FSh). This variable accounts for the net shape change in a breathing cycle [24]; this is, the magnitude of shape variation attributed just to breathing kinematics for each subject in each condition.

Finally, the null hypothesis for shape (H3_shape_) predicts no differences in shape between EX during QB and EX during REC and between IN during QB and IN during REC. We compared the corresponding medians of PC1 and PC2 scores which accounted for approximately 50% of total shape variance.

All the analyses were carried out in R environment (R v. 4.2.0 and RStudio v. 1.4.1717). Statistical analyses were made through R packages rstatix [25], stats [26] and car [27], and all the 3DGM analysis and three-dimensional warping visualizations were made using R packages geomorph [23], Morpho [28], Rvcg [29] and rgl [30]. Electronic supplementary material, video (SV) material, was created through EVAN Toolbox software [31].

### 2.6. Three-dimensional mesh warping and visualization

The concept of three-dimensional warping consists of the deformation of a three-dimensional mesh, given a set of reference coordinates (original coordinates of the selected three-dimensional mesh) and a corresponding set of target coordinates (i.e. the theoretical shape corresponding to a set of PC scores). Prior to OEP recording, the three-dimensional surface shape of the STA group torso with the attached 89 markers (= landmarks) was collected using a structured-light three-dimensional surface scanner (Artec Eva, www.artec3d.com). This was done in a standardized position, standing upright on a turning table in quiet breathing with the arms and hands placed on the hips. The landmarks were digitized in one three-dimensional model of the sample using the lhpFusionbox software (https://anatbiomecaorgano.ulb.be/biomechanics/The_lhpFusionBox.html), with the purpose of using it for the shape visualizations of the statistical analyses. Afterwards, three-dimensional warping was used to graphically represent the theoretical shape at the maximum and minimum principal components of the PCAs and to represent the mean shape of each posture and condition.

## 3. Results

### 3.1. Principal components analysis of both postures and conditions together

**Figure 3.**
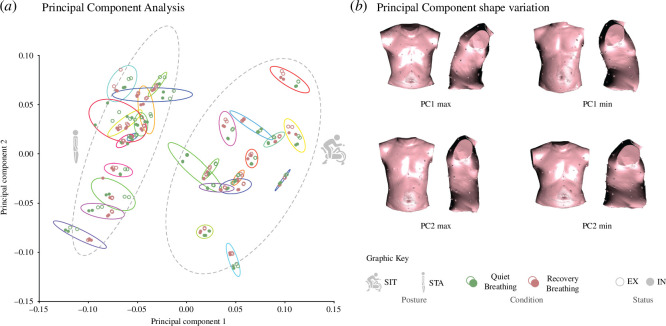
PCA explaining the shape variance along PC1 and PC2 (GPA of SIT and STA together). (*a*) Scatterplot of the PC scores of each subject and condition. (*b*) Patterns of shape variation associated with PC1 and PC2. Notice the effect of motion (EX–IN differences) along both PCs. Group ellipses (dashed lines) characterize each subject (solid lines) with a confidence interval of 0.9. PC1 (35.04%) and PC2 (18.35%) accounted for 53.38% of total shape variance.

Figure 3 shows a PCA over the covariance matrix of the Procrustes coordinates of the IN and EX observations of each subject and breathing condition (GPA of SIT and STA together). PC1 and PC2 of the resulting PCA accounted for 53.38% of the total shape variance. This PCA ordered the different configurations in a morphospace that included variation related also to CW motion, and not only to posture and CW shape. Each subject is represented by different shape configurations that consider the breathing condition (QB or REC) and instants (EX or IN).

The joint study of both groups in the PCA allows us to describe the postural variation (inclined, sitting on the ergometer in SIT or upright standing in STA), breathing conditions (QB or REC) and instants (EX or IN) from a common statistical space, making the subjects comparable throughout the different PCs (common shape variation). The first PC (35.04%) clearly explains postural variation (figure 3) and ranges from an extended posture of the trunk (negative values of PC1, STA) to an inclined posture towards positive values of PC1 (SIT). SIT and STA show a similar variation range along PC1 (electronic supplementary material, table S1), indicating there is the same amount of postural variation for sitting and standing postures.

The second PC (18.35%) shows the principal shape variation of the trunk that is not related to posture and common to both SIT and STA (figure 3). This variation ranges from tall, flat and narrow trunks towards positive values, to short, deep and wide trunks towards negative scores. Both SIT and STA postures show similar ranges of trunk shape variations (see electronic supplememtary material, table S1).

Besides trunk shape, PC1 and PC2 are also collecting information about breathing motion. As observed in figure 3, the PCA slightly separates EX and IN in both PCs. This means that breathing motion, expressed as change in shape between EX and IN of the same subject and breathing condition, is expressed along PC1 as a breathing-related motion component of change in postures and along PC2 as a breathing-related motion component of change in torso shape.

### 3.2. Size analyses

**Figure 4.**
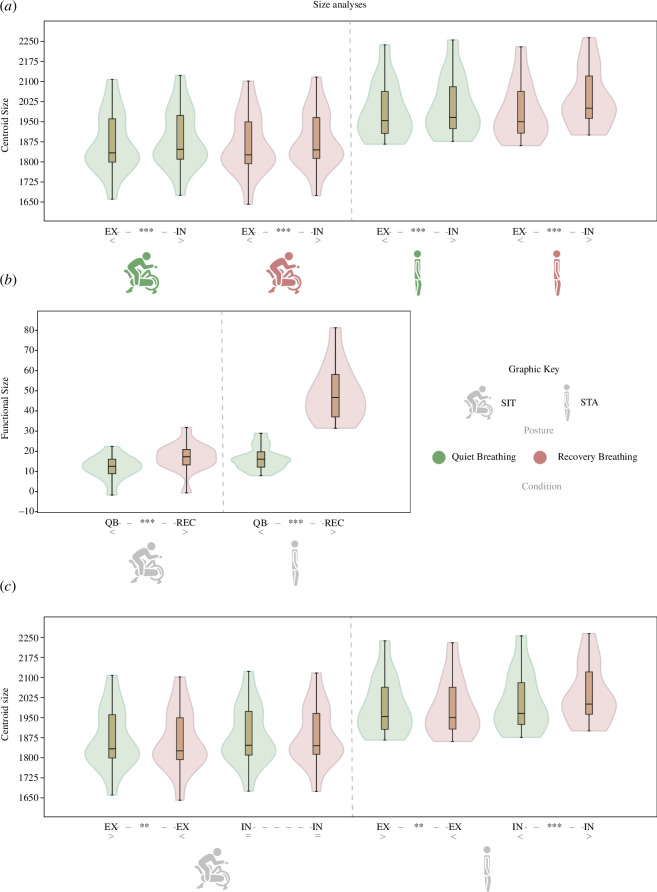
Violin and box plots of the three size analyses with statistical significance of the comparisons. (*a*) H1_size_: CS of EX and IN configurations in each posture and breathing condition. (*b*) H2 _size_: functional size, which measures the amount of size differences between EX and IN of QB and REC in each of the postures. (*c*) H3 _size_: CS of EX and IN configurations between breathing conditions. Comparisons below the violin plots indicate statistical differences between the compared groups. *p*‐value: ***<0.001; **<0.01 (for statistics of size analyses, see electronic supplememtary material, S2–S4).

The median of IN configurations was significantly higher (*p *< 0.01) than that of EX. This pattern was found for each posture and condition. Thus, the null model of H1_size_ is rejected (figure 4*a*, comparisons below the violin graphs, electronic supplementary material, table S2).

Regarding functional size (H2_size_), the results (figure 4*b*; electronic supplementary material, tableS3) show significantly lower FS in QB than in REC in both postures. In addition, the larger change in FS owing to exercise (i.e. FS_QB_ – FS_REC_) is observed in STA.

In order to understand these differences in functional size between conditions depending on the experiment, we tested H3_size_ (figure 4*c*; electronic supplementary material, S4). The results showed that CS of EX was statistically significantly larger for the QB condition than for the REC condition in both SIT and STA. Thus, during REC, expiration was greater than during QB. However, CS of IN of SIT showed no differences depending on the breathing condition, while in STA CS of IN was statistically significantly smaller for the QB condition than for the REC condition. Thus, the null model of H3_size_ is rejected for all experiments, except for IN in SIT.

### 3.3. Shape analyses

**Figure 5.**
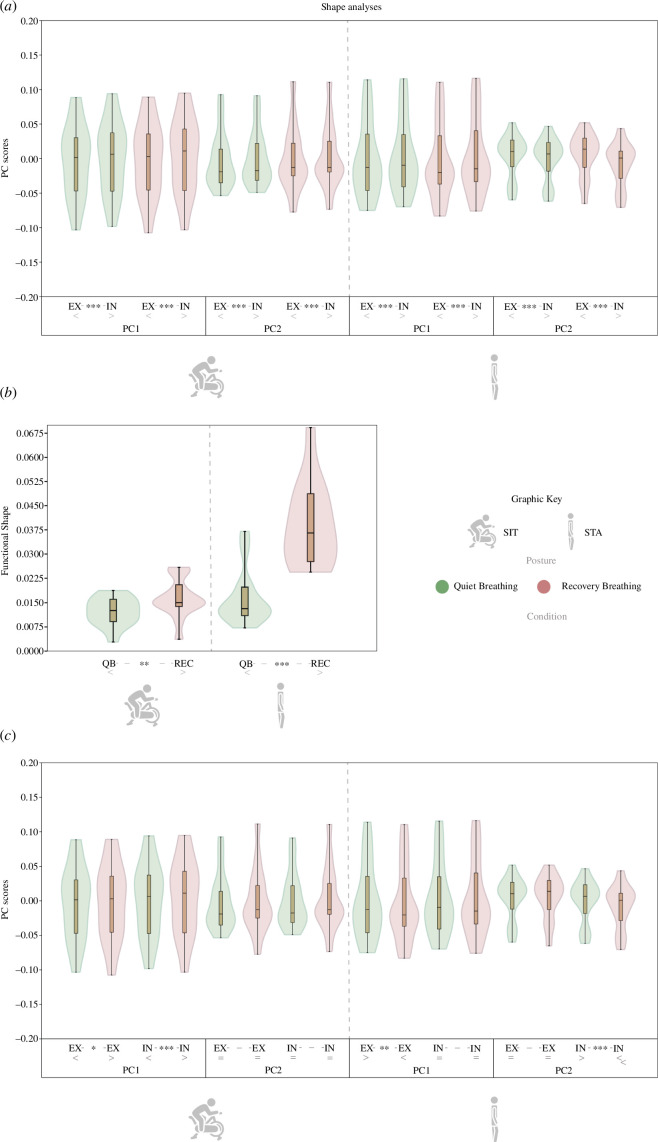
Violin and box plots of the three shape analyses with statistical significance of the comparisons. (*a*) H1 _shape_: PC1 and PC2 scores of EX and IN configurations in each posture and breathing condition. (*b*) H2 _shape_: functional shape (Procrustes distance), which measures the amount of shape (and motion) differences between EX and IN of QB and REC in each of the postures. (*c*) H3 _shape_: PC1 and PC2 scores of EX and IN configurations between breathing conditions in each posture. *p*-value: ***<0.001, **<0.01, *<0.05 (statistics of shape analyses at electronic supplementary materials, S5–S7). Comparisons below the violin plots indicate statistical differences between the compared groups.

**Figure 6.**
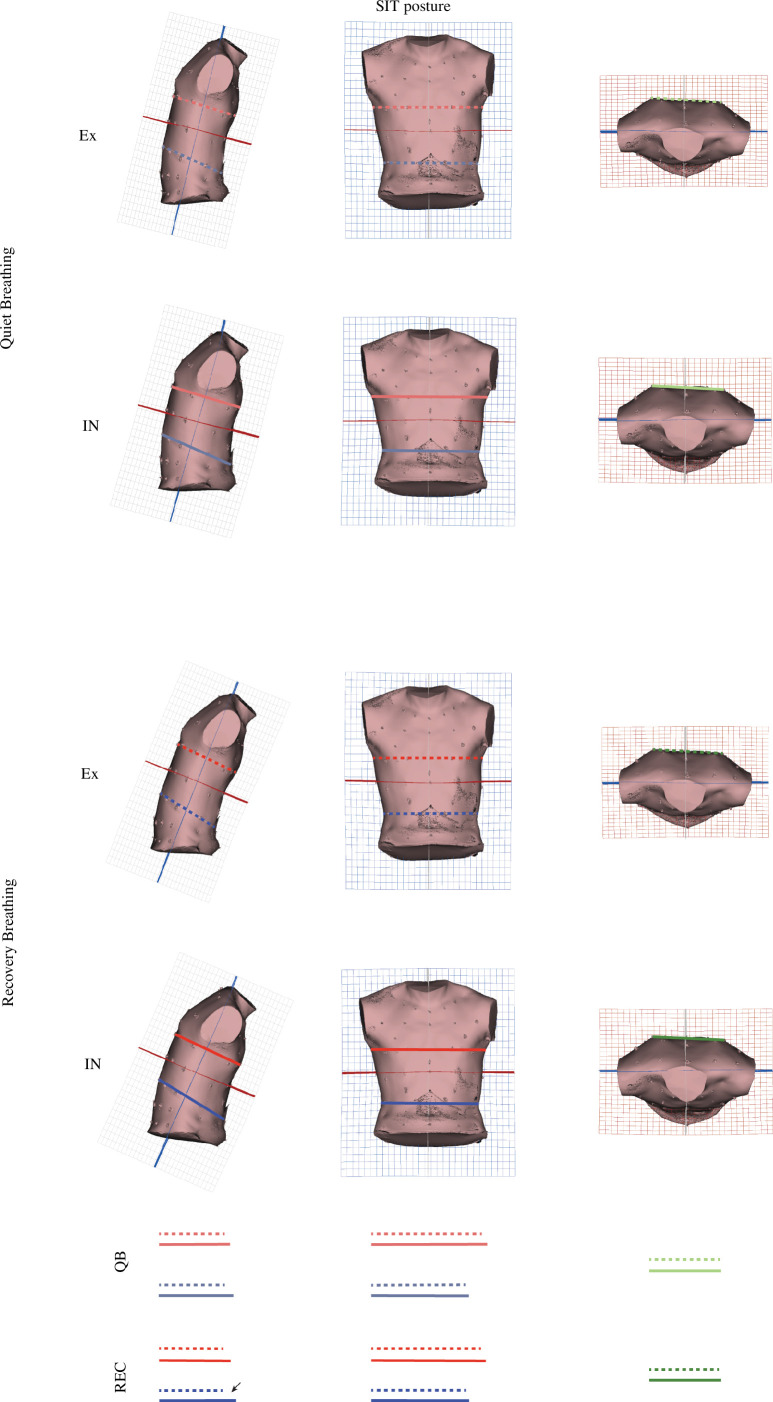
Three-dimensional warps of mean EX and mean IN shape configurations at both QB and REC conditions in PC1 and PC2 shape space of SIT PCA (electronic supplementary material, S2, electronic supplementary material, Video). Lines are for aiding the graphical explanation of results. Dashed line: expiration; solid line: inspiration; red line: thorax depth/width; blue line: abdomen depth/width. Arrows indicate stronger differences.

**Figure 7.**
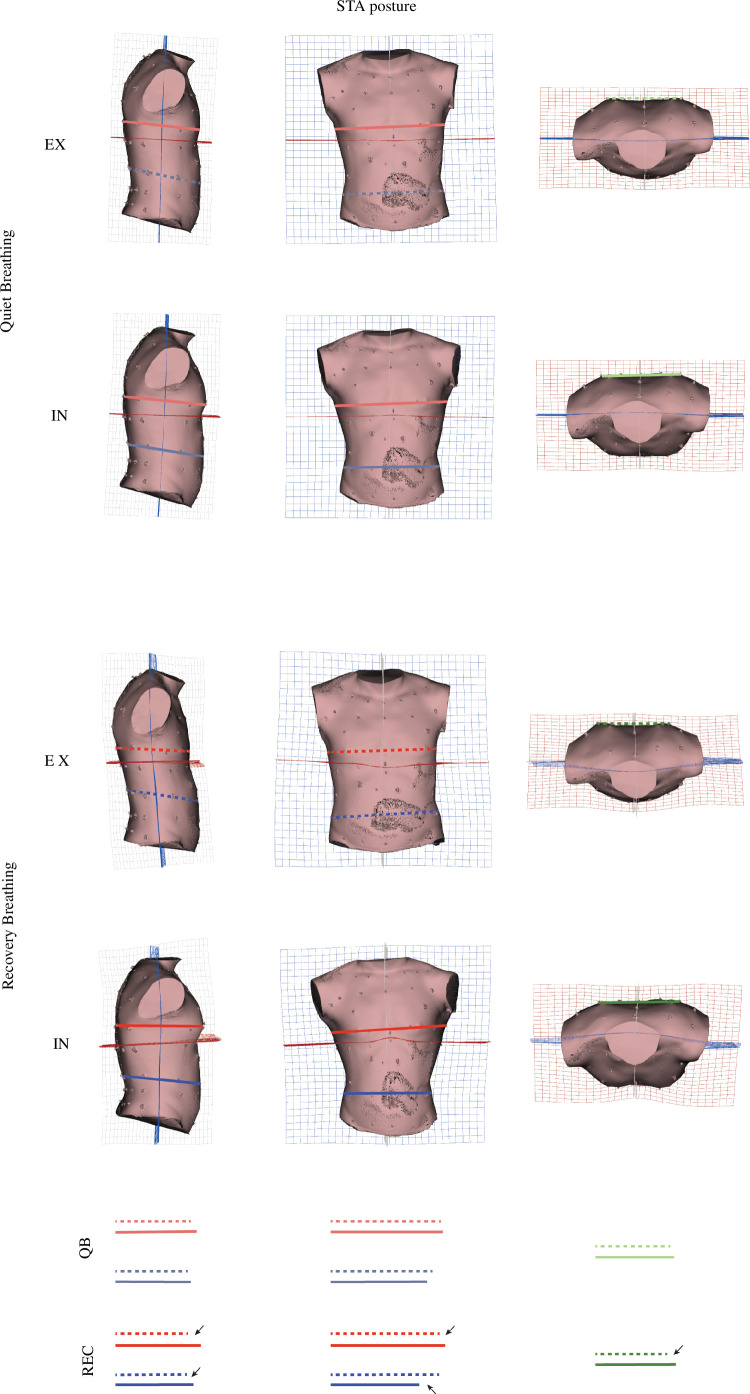
Three-dimensional warps of mean EX and mean IN shape configurations at both QB and REC conditions in PC1 and PC2 shape space of STA PCA (electronic supplementary material,figureS3, electronic supplementary material,video). Lines are for aiding graphical explanation of results. Dashed line: expiration; solid line: inspiration; red line: thorax depth/width; blue line: abdomen depth/width. Arrows indicate stronger differences.

PC scores (separate SIT and STA GPAs; electronic supplementary materials, S2, S3) show a significant difference between IN and EX configurations in all conditions (figure 5*a*, electronic supplementary material, S5). These results reject H1_shape_ and are shown in figures 6 and 7.

For SIT posture, the differences in shape between EX and IN are associated with dorsoventral deformation of the torso. Specifically, TPS grid deformations (figure 6) in QB condition show greater shape variation in the abdomen (see electronic supplementary material, video, QB SIT). Also, a significantly more pronounced abdominal motion (electronic supplementary material, video, REC SIT) is observed in REC condition.

In STA posture, we find slightly different patterns. Greater shape variation on the upper CW is observed during QB with cranial and ventral displacement over inspiration (electronic supplementary material, video, QB STA).

Another feature in STA (not observed in SIT) relates to the spine curvature: thoracic kyphosis and lumbar lordosis are respectively greater and lower during EX. TPS grid deformations and warped surfaces show (electronic supplementary material, video, REC STA) that this pattern is increased in REC condition compared with QB.

These statistical analyses reject the H1_shape_ and together with their graphical visualizations support that breathing motion during QB and REC generates significant shape differences between EX and IN for both postures.

Results also reject H2_shape_ and reveal significant differences in FSh between QB and REC (see figure 5*b* and electronic supplementary material, tableS6). For both postures, QB showed less FSh than REC, but the differences between medians were larger in the STA posture.

Finally, H3_shape_ is also rejected by displaying statistically significant differences in the shape (PC1 and PC2 scores) of both EX and IN configurations between QB and REC. In SIT posture, IN/EX shape differences are observed along PC1 while in STA differences are observed along PC1 and PC2 (see figure 5*c*, electronic supplementary material, tableS7, electronic supplementary materials, figureS2, S3). These differences are graphically explained through figures 6 and 7, which describe the shape and motion variation associated with each PC. In SIT posture, QB shows less dorsoventral motion of the CW and higher torso flexion than REC. In STA posture, QB shows less dorsoventral motion of the CW, less abdominal expiratory action (PC1, electronic supplementary material, figureS3) and less thoracic contribution to inspiration (PC2, electronic supplementary material, figureS3) than REC.

The pattern found in SIT posture is different from the one of the STA posture. The former shows a higher amplitude of inspiratory motion and less marked expiratory motion in REC compared with QB, while the latter shows a greater range of motion both in inspiration and expiration (see mean respiratory vectors of electronic supplementary materials, figure S2, S3).

## 4. Discussion

The present results illustrate the potential of the innovative combination of 3DGM and OEP for evaluating respiratory patterns in various conditions of posture and/or physical exercise. We first explored overall shape variation in relation to these factors. Then, we addressed hypotheses about the changes in size and three-dimensional shape of the CW, (i) between quiet spontaneous breathing at rest (QB) and spontaneous breathing during the recovery phase immediately after intense exercise (REC), and (ii) between standing (STA) and sitting (SIT) on the cycle ergometer.

This study presents a pioneer methodology and it is essential to acknowledge its preliminary nature. Aspects such as limited sample size may require to interpret statistical results with caution. Additionally, results may vary depending on BMI or sexual dimorphism, which are not controlled for in the study. Given these considerations, the results should be carefully interpreted. Nonetheless, this study provides valuable insight into the potential of the developed methodology in the context of breathing kinematics, being able to detect even subtle differences in quiet breathing. Thus far, the methodological approach to the study of CW kinematics consisted of volume computations of its different compartments, and calculation of distances between markers (diameter calculation) for the determination of breathing pattern [4,32]. Here, we propose 3DGM as a novel and accurate method to measure the shape and motion of the CW, which, combined with other standard analyses will provide a better understanding of how respiratory mechanics work. In fact, breathing patterns and postural factors were detected easily in the general analysis of shape variation (full PCA, figure 3), which, in addition, overlapped partially with variations of the proper CW shape of the individual subjects. As expected, the most important factor in CW shape variation was posture (PC1), while the second most important factor was trunk shape (PC2). Interestingly, in this morphometric subspace, respiratory patterns showed up slightly oblique to both PCs, meaning that shape changes related to respiratory function mimic some aspects of variation related to body shape and posture. This suggests that trunk morphology (e.g. flat and narrow thoraces or deep and wide thoraces) and respiratory motion are intricately related following parallel patterns of variation.

This can be explained by looking at the shapes associated with PC1 and PC2. While sitting on the ergometer *per se* implies a slight anterior flexion of the torso (PC1 max), standing upright *per se* implies a relative extension of the trunk (PC1 min). Respiratory motion can show shape changes similar to the postural related ones. For instance, inspiration is related to an upper trunk expansion, and increasing the vertical and ventral dimensions related to rib rotation [33,34] during spontaneous recovery. Expiration in REC can require anterior flexion of the CW owing to abdominal muscle contraction [2] and makes the torso flatter and narrower, which are also the natural dimensions of variation in subject-specific torso shape. However, while this three-dimensional shape analysis revealed similarities among posture, body shape and kinematics together with individual variations, it also indicated that a more specific analysis of breathing patterns and their changes from QB to REC would require controlling for posture as suggested by previous research [6,35–37].

### 4.1. Different breathing modes in terms of size

Statistically significant differences in size (CS) at rest suggest the ability of the 3DGM toolkit to estimate subtle variations while breathing at tidal volume. This allows for estimating the effect of specific respiratory conditions, such as before and after exercise. These results are in line with previous research based on volumetric measurements [1,3]. This was expected, however, because CS is based on the three-dimensional coordinates that are also used for volume computations. On similar grounds, we interpret the fact that functional size is greater in recovery breathing than in quiet breathing reflecting the increase in tidal volume in the context of exercise [1,2].

Nevertheless, it is interesting to observe that functional size in STA is greater than in SIT, which may be owing to the fact that in SIT the kinematics of the thorax could be more constrained owing to the more flexed posture (figure 3). In fact, in STA we found QB-REC differences in size both in EX, potentially owing to a variation during expiration and in IN, while in SIT no differences were observed in IN.

### 4.2. Different breathing modes in terms of three-dimensional shape

Although shape studies of breathing kinematics have not yet been carried out in the context of 3DGM, it is still possible to compare our shape results with respect to volumetric studies that contemplate the relative sizes of CW compartments. This is because the variation of the relative sizes of the compartments corresponds to variation in the overall shape of the entire CW.

During QB, it is assumed that motion is mainly driven by the contraction of inspiratory muscles, with a higher contribution of the inspiratory rib cage muscles compared with the diaphragm [1,3]. Exercise breathing changes the ‘role’ of the diaphragm, which changes from ‘pressure generator’ at rest to ‘flow generator’ [3], leading to potential diaphragmatic fatigue [38]. Simultaneously, exercise increases the recruitment of the respiratory rib muscles for inspiration and abdominal muscles for expiration in order to increase tidal volume and therefore to ventilate [1,3,38,39].

Results show that in SIT the respiratory shape changes during QB are mainly located along the anteroposterior axis of the entire torso (slightly greater change at the abdomen). These results are in line with previous research on breathing at rest during sitting that displayed primarily ventral displacement of the abdomen in contrast with the cranial and lateral movements of the rib cage [40]. During REC, there is an increase in the intensity of this breathing pattern.

Interestingly, in QB of STA, a greater variation of the anteroposterior diameter of the entire torso than in SIT was observed, with a more marked expansion located particularly at the thoracic part. Previous research mainly explored the effect of various sitting postures [4] or between sitting and supine/prone postures [6,35–37]. Surprisingly, only one study compared results between sitting and standing positions and it reported no significant differences in thoracoabdominal motion between these postures during QB [6]. Our results suggest a larger contribution of the thoracic part in STA that could be related to the greater abdominal wall tension and intra-abdominal pressure in standing when compared with sitting [41,42].

During REC, this pattern becomes stronger in STA, where the thoracic part becomes markedly anteroposteriorly deeper and mediolaterally wider than in SIT. This higher thoracic expansion, which leads to greater sizes at IN in STA group, highlights the potential interest of using both CS and shape in biomechanical analysis. Size analysis only gives information about volumetric changes, while shape analysis informs us about thoracic upper spine extension, informing us about the kinematics behind the volumetric change. Also, during REC of STA we can observe shoulder girdle rotation on coronal view. In SIT, we do not find any shape changes in this region, which could be owing to the postural constraint of the handlebars stabilizing upper limbs on the cycle ergometer.

In terms of breathing pattern change from QB to REC, the analysis of the morphometric subspace shown by the separate groups of PCAs is surprising (figure 5; electronic supplementary material, figureS2, S3). PCA is reflecting the different breathing pattern related to each posture. When comparing QB and REC mean respiratory vectors, the SIT group shows their spatial relationship as a moderate translation along PC1 mainly. This means a greater inspiratory motion and similar expiratory motion in REC. On the other hand, the STA group shows a greater module of the REC respiratory vector compared with QB, which implies not only greater inspiratory but also greater expiratory motion. We suggest that these differences in motion may be related to postural constraints derived from sitting on the ergometer. At SIT posture, during both QB and REC, we can only observe thoracic motion in the ventral direction, while in STA posture, thoracic motion explores cranial, lateral and ventral directions. Such differences can only be interpreted anatomically by shape analyses. They are in line with the results presented by Crawford *et al.* [43], which indicated that the pattern of volume change of the ribcage in subjects seated on a cycle ergometer was similar to that of quiet breathing. Nonetheless, this does not refute the findings of De Groote *et al.* [40]; instead, we believe it may reflect the importance of the upper limb positioning during breathing, which should be considered when interpreting breathing mechanics and physiology during cycling exercise.

The relation between posture and breathing kinematics is also interesting in light of the compartmental approach of the CW [44]. When comparing the rib cage and the abdomen as separate compartments, we can observe that in SIT, greater differences in motion are found at the level of the abdomen while, in STA, differences are concentrated on the rib cage. When considering a compartmental approach, different postures affect breathing pattern, but maximal mechanic efficiency can be achieved through different volume displacements at different levels of the CW. Our results are probably showing the adaptability of the CW system to changes with posture. Thus, SIT posture can affect the mechanical properties of the rib cage, possibly owing to constraints exerted by the shoulder girdle and spine flexion. To compensate for these potential constraints, the abdominal region would gain importance in breathing pattern. In STA, less postural constraint on motion would result in torso extension, elevation and expansion of the rib cage compartment.

Overall, these findings underscore the complexity of the respiratory function and its close link to body posture. This brings up the need to understand how those interact, with utility not only in scientific research but also potentially in different clinical settings. The present study supports the use of 3DGM as an innovative and powerful approach to the study of breathing mechanism. We have shown that CS analysis connects well to what has been suggested on lung volume variations, while shape connects mainly to specific anatomically relevant details of the breathing behaviour.

## Data Availability

The data supporting the findings of this study can be found available as a supplementary file including the list of three-dimensional coordinates of the chest wall of the subjects [45].
